# Macrophages in non-infectious uveitis: cell origin, disease stage, evidence provenance, and therapeutic maturity

**DOI:** 10.3389/fimmu.2026.1958864

**Published:** 2026-09-28

**Authors:** Hao Yue, Jing Lu, Yasha Zhou, Li Xiao, Ying Deng, Qinghua Peng, Yijing Yang

**Affiliations:** 1 Faculty of Traditional Chinese Medicine, Hunan University of Chinese Medicine, Changsha, China; 2 Institute of Ophthalmology and Otolaryngology of Chinese Medicine, Hunan University of Chinese Medicine, Changsha, China; 3 Changsha Centre for Innovation in Traditional Chinese Medicine Techniques for the Prevention and Treatment of Retinal Diseases and Visual Function Protection, Hunan University of Chinese Medicine, Changsha, China

**Keywords:** experimental autoimmune uveitis, macrophage, microglia, monocyte-derived macrophage, non-infectious uveitis, single-cell RNA sequencing, therapeutic target, translational immunology

## Abstract

Non-infectious uveitis (NIU) comprises a heterogeneous group of immune-mediated disorders in which myeloid cells influence leukocyte entry, inflammatory amplification, tissue injury, resolution, and relapse. The conventional dichotomy of classically activated (M1) and alternatively activated (M2) macrophages does not adequately capture this functional diversity. This narrative review organizes the evidence along three complementary dimensions: cell origin, disease stage, and evidence provenance; therapeutic maturity is evaluated separately. Experimental data support distinct roles for resident retinal microglia, retinal perivascular macrophages, vitreal hyalocytes, uveal macrophages, and recruited monocyte-derived macrophages. Microglial major histocompatibility complex class II signaling appears to amplify locally restimulated T-cell responses, whereas depletion with a broad colony-stimulating factor 1 receptor inhibitor cannot be interpreted as microglia-specific. Perivascular macrophages are positioned to regulate antigen presentation and leukocyte entry at the blood-retinal barrier, but their contribution to human NIU remains poorly defined. Recruited monocyte-derived macrophages contribute to inflammatory amplification in experimental autoimmune uveitis (EAU), while reparative and persistent states remain stage dependent. Human ocular fluid and single-cell studies provide direct observations of inflammatory myeloid states but rarely establish ontogeny or causality. In ankylosing spondylitis-associated acute anterior uveitis, cluster of differentiation 14-positive and intercellular adhesion molecule 1-high (CD14+ICAM1-high) monocytes showed trained-immunity-like transcriptional, functional, epigenetic, and metabolic features, with a computationally inferred transition toward macrophage states in aqueous humor. Clinically, tumor necrosis factor blockade provides the strongest therapeutic evidence. Janus kinase inhibition has randomized phase 2 support, whereas interleukin-6, interleukin-1, P2X purinoceptor 7, chemokine, inflammasome, metabolic, and reparative strategies remain less mature or are not myeloid specific. Progress will require phenotype- and stage-matched studies that combine longitudinal ocular and blood sampling with cell-resolved biomarkers, target engagement measures, and experiments involving delayed treatment in complementary animal models.

## 1 Introduction

Non-infectious uveitis (NIU) comprises immune-mediated inflammatory disorders affecting the uvea, retina, retinal vessels, vitreous, and adjacent ocular tissues. Visual loss may result from an acute inflammatory episode, but it also reflects macular edema, occlusive vasculitis, photoreceptor and retinal pigment epithelium injury, and cumulative structural damage during recurrent disease. Autoreactive cluster of differentiation 4-positive (CD4+) T cells and cytokine networks are central to pathogenesis, and experimental autoimmune uveitis (EAU) remains the principal platform for causal investigation. However, anatomical classification, systemic association, and conventional activity measures do not fully explain why clinically similar episodes range from rapid resolution to irreversible neurovascular injury or relapse. This heterogeneity complicates mechanistic interpretation and the selection of stage-appropriate therapy (1–7).

Macrophage-lineage cells connect peripheral autoimmunity with tissue-specific outcomes without replacing the established T-cell-centered model. Resident retinal microglia, retinal perivascular macrophages, vitreal hyalocytes, uveal macrophages in the choroid, iris, and ciliary body, choroidal dendritic cells, and recruited monocyte-derived macrophages differ in developmental origin, anatomical access, turnover, and local exposure. These distinctions affect their functions during leukocyte entry, inflammatory amplification, debris clearance, barrier repair, and persistent remodeling. Most causal evidence for this sequence comes from EAU, whereas direct attribution to defined myeloid subsets in patients remains limited (8–26).

The relative importance of these mechanisms is likely to vary among clinical phenotypes. Acute human leukocyte antigen B27 (HLA-B27) anterior uveitis primarily involves the iris, ciliary body, and blood-aqueous barrier; Behcet’s uveitis often includes occlusive retinal vasculitis and neutrophil-rich inflammation; juvenile idiopathic arthritis (JIA)-associated uveitis is typically chronic and anterior; and birdshot chorioretinopathy and sarcoid uveitis preferentially affect posterior or granulomatous compartments (1–7). A finding from EAU induced by active immunization therefore should not be assigned equal importance across all forms of NIU. Likewise, the terms “ocular macrophage” and “classically activated (M1)/alternatively activated (M2) macrophages” are too broad to define ontogeny, anatomical niche, disease timing, or function. Each mechanistic claim should identify the relevant cell population, compartment, disease stage, experimental system, and level of human support.

This review addresses that problem through a three-axis framework based on cell origin, disease stage, and evidence provenance. Evidence sources are reported with non-ordinal tags because human intervention, patient-derived observation, causal model perturbation, associative model data, and cross-disease context answer different questions. Resident microglia, retinal perivascular macrophages, vitreal hyalocytes, uveal macrophages, and recruited monocyte-derived macrophages are mapped from priming and neurovascular entry through amplification, resolution, and relapse. **Figure 1** places these populations within clinical anatomy and model heterogeneity, whereas **Figure 2** aligns cell origin and disease stage with explicit provenance indicators. Therapeutic maturity is assessed separately to avoid equating mechanistic plausibility with clinical validation.

**Figure 1:**
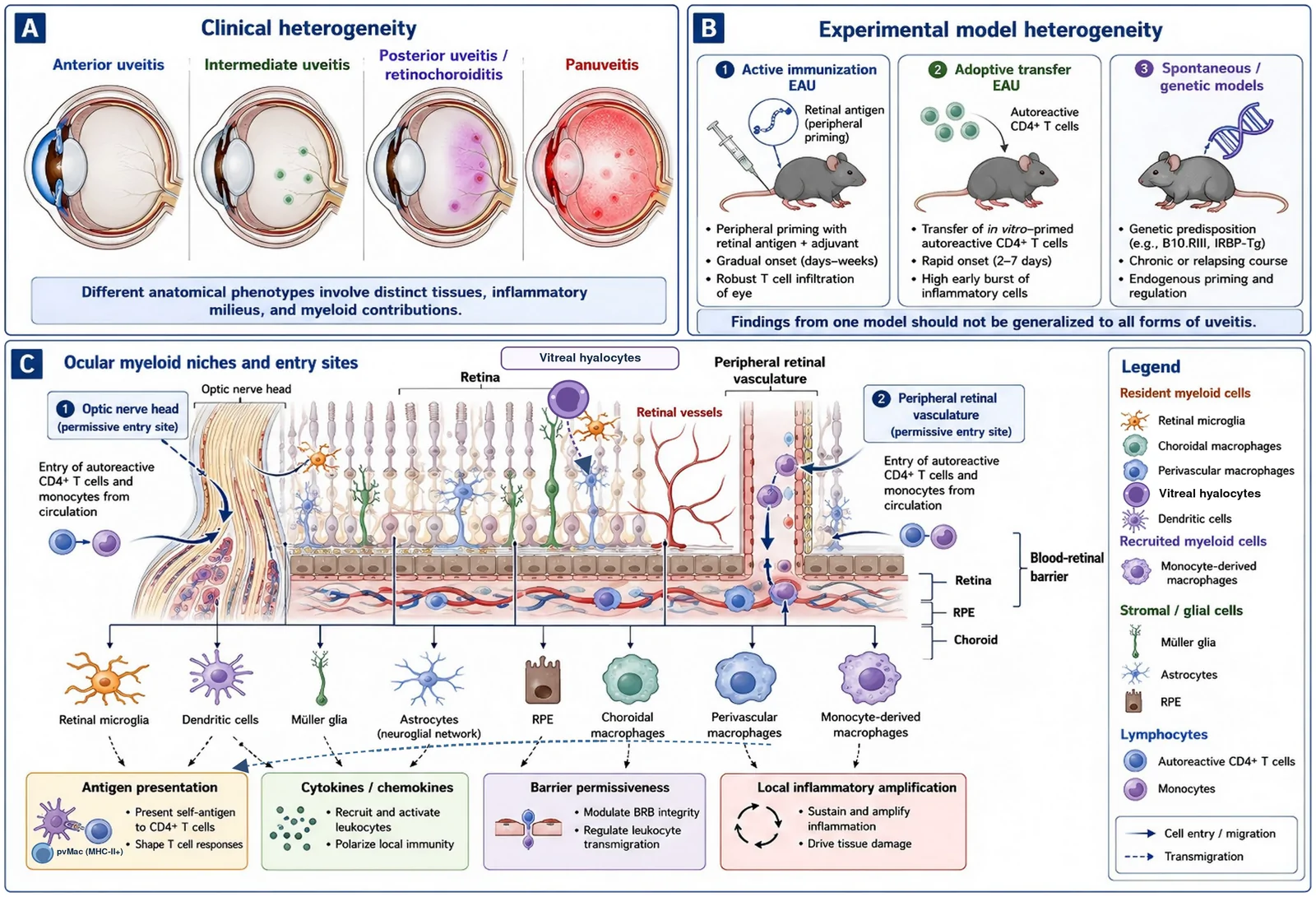
Clinical and experimental model heterogeneity, compartment-resolved ocular myeloid niches, and principal neurovascular entry sites in non-infectious uveitis. Retinal perivascular macrophages are shown as a distinct venular population at the blood-retinal barrier and are included in the antigen presentation module because of their phenotype enriched for major histocompatibility complex class II (MHC II). Vitreal hyalocytes are shown separately at the vitreoretinal interface. Uveal macrophages and choroidal dendritic cells are not grouped with retinal perivascular macrophages under a generic “border-associated macrophage” label. Findings from one experimental autoimmune uveitis (EAU) or ocular neuroinflammation model should not be generalized to all forms of uveitis. BRB, blood-retinal barrier; EAU, experimental autoimmune uveitis; MHC II, major histocompatibility complex class II; pvMac, retinal perivascular macrophage; RPE, retinal pigment epithelium.

**Figure 2:**
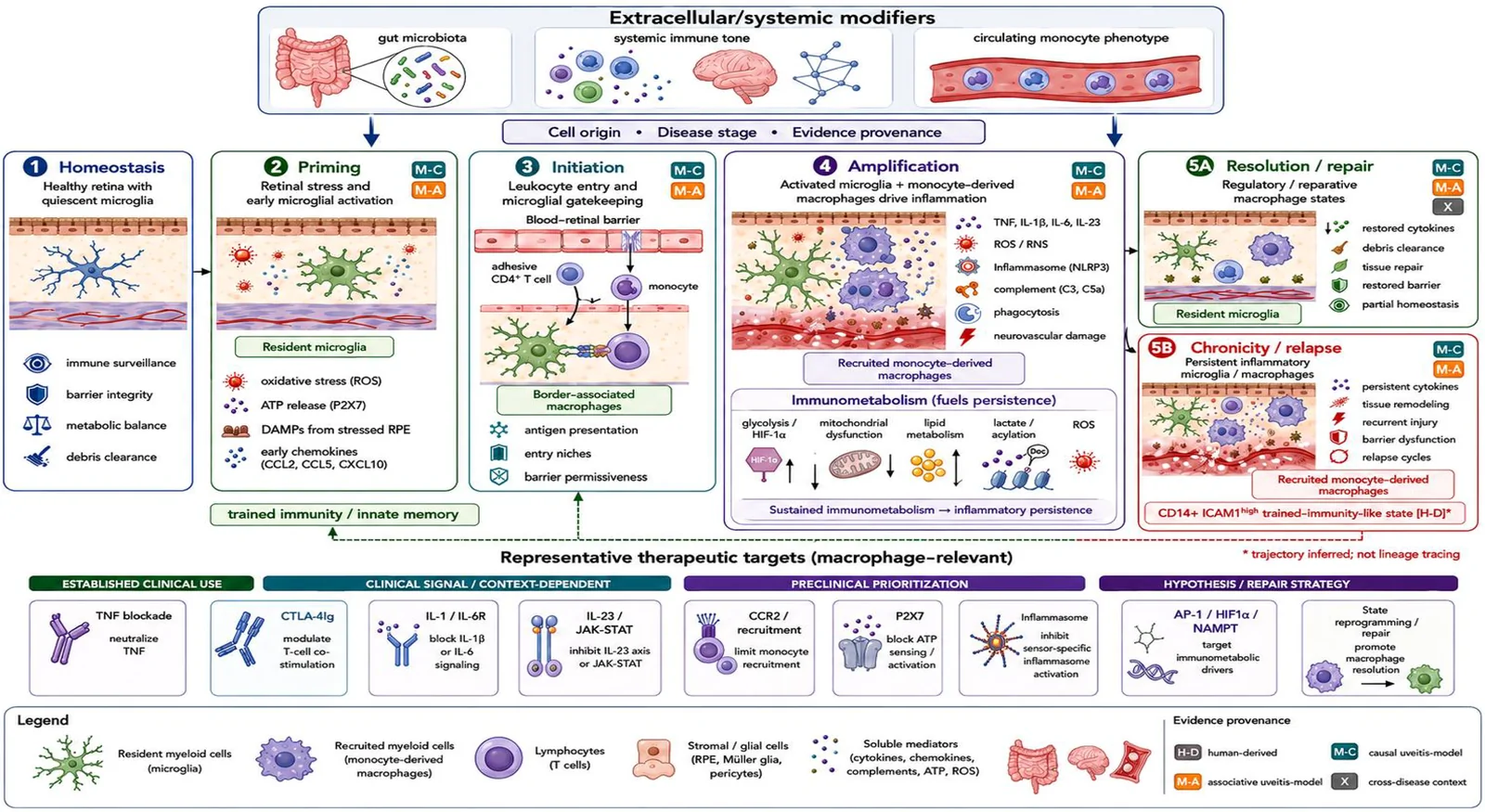
Stage-based map of macrophage biology and therapeutic maturity in non-infectious uveitis. The horizontal sequence extends from homeostasis and priming through initiation, amplification, resolution or repair, and chronicity or relapse. Cell origin labels distinguish resident retinal microglia, retinal perivascular macrophages, and recruited monocyte-derived macrophages. Evidence provenance badges indicate human-derived evidence (H-D), causal evidence from uveitis models (M-C), associative evidence from uveitis models (M-A), and cross-disease context (X). Therapeutic maturity is displayed separately as established clinical use, clinical signal or context-dependent evidence, preclinical prioritization, or a hypothesis or repair strategy. The proposed transition from circulating trained-immunity-like monocytes to ocular macrophage states represents trajectory inference rather than lineage tracing. AP-1, activator protein 1; C3, complement component 3; C5a, complement component 5a; CCR2, C-C chemokine receptor type 2; CTLA-4-Ig, cytotoxic T-lymphocyte-associated protein 4 immunoglobulin; HIF-1α, hypoxia-inducible factor 1 alpha; IL, interleukin; JAK–STAT, Janus kinase–signal transducer and activator of transcription; NAMPT, nicotinamide phosphoribosyltransferase; NLRP3, NOD-like receptor family pyrin domain-containing 3; P2X7, P2X purinoceptor 7; RNS, reactive nitrogen species; ROS, reactive oxygen species; TNF, tumor necrosis factor.

## 2 Evidence synthesis and principal findings

### 2.1 Salient patterns in the evidence matrix

**Table 1** shows that evidence strength depends on both the biological question and the source of the data. In EAU, the clearest causal findings concern colony-stimulating factor 1 receptor (CSF1R)-dependent resident myeloid cells, microglial major histocompatibility complex class II (MHC II) signaling, monocyte recruitment in selected models, P2X purinoceptor 7 (P2X7) signaling, and sensor-specific inflammasome pathways. Human studies provide direct evidence of ocular cytokine patterns, cell-resolved inflammatory states, and treatment responses, but they rarely establish the developmental origin of ocular macrophages or prove that a particular myeloid state causes tissue injury. Evidence is least mature for vitreal hyalocytes, subtype-specific uveal macrophages, reparative macrophage states, and the persistence of trained-immunity-like programs through remission and relapse.

**Table 1:** Cell origin, disease stage, principal function, evidence provenance, and remaining inference gaps.

Cell origin	Disease stage	Principal function	Evidence provenance	NIU interpretation/critical gap
Resident retinal microglia	Homeostasis/priming	Surveillance; early chemokine and antigen presentation programs	M-C and M-A: depletion, activation timing, longitudinal profiling (9, 78)	Inference: CSF1R-dependent resident myeloid cells are required in EAU; microglia-specific initiation remains unresolved. Gap: longitudinal, subset-resolved human evidence.
Resident retinal microglia	Initiation/entry	Integrate ATP, glial, and RPE cues; influence leukocyte entry	M-C: depletion and pathway perturbation (9, 11, 59)	Inference: supported in EAU; direct human lineage attribution lacking. Gap: cell-resolved validation in defined NIU phenotypes.
Perivascular macrophages	Entry	Spatial control of vascular infiltration	M-A: mouse ocular neuroinflammation and trafficking studies (18, 43)	Inference: model-supported role at sites of leukocyte entry; not yet demonstrated in human retinal vasculitis. Gap: human spatial and functional confirmation.
Uveal macrophages and choroidal dendritic cells	Entry/amplification	Functions at the RPE–choroid and anterior segment interfaces	M-A plus X: anatomical and broader myeloid evidence (28, 34, 108)	Inference: anatomical and cross-disease evidence supports niche-specific hypotheses. Gap: direct tissue and functional evidence within defined NIU phenotypes.
Monocyte-derived macrophages	Amplification	Cytokine, oxidative, and phagocytic programs	M-C and M-A: EAU kinetics and perturbation (15, 16, 90)	Inference: causal/therapeutic support in models; ocular lineage unresolved in patients. Gap: paired ocular and blood lineage and target engagement data.
Microglia and recruited macrophages	Tissue injury	P2X7 sensing; sensor-specific inflammasome and pyroptosis pathways	M-C with X background: EAU genetic/pharmacological studies and retinal review (59–64, 66)	Inference: sensor-specific effects in EAU; human ocular confirmation lacking. Gap: biomarker of active sensor signaling and safety window.
Metabolically activated myeloid states	Amplification/persistence	HIF-1α, glycolysis, lactylation, NAMPT, and AP-1 programs	M-C/M-A; limited human peripheral support (54, 88, 107, 109–112)	Inference: pathways are supported; dominant ocular human cell remains uncertain. Gap: prospective cell-resolved biomarkers and delivery.
Regulatory/reparative states	Resolution/repair	Inflammatory restraint; debris clearance; neural and barrier support	M-C plus X: EAU and retinal injury models (67–69)	Inference: repair potential supported, but human state definition is lacking. Gap: definition beyond M1/M2 and human validation.
Persistent circulating and ocular myeloid states	Chronicity/relapse	Trained-immunity-like programs; incomplete reprogramming	H-D, M-A, and X: patient single-cell/functional data, EAU, and cross-disease context (62, 73–78)	Inference: patient-linked trained-immunity-like state; transition and relapse causality remain inferred. Gap: longitudinal paired sampling through remission and relapse.

Evidence provenance is reported as complementary source information rather than as an ordinal rank. H-I denotes human intervention; H-D, human-derived observational or functional evidence; M-C, causal perturbation in a uveitis model; M-A, associative or descriptive evidence in a uveitis model; and X, cross-disease contextual evidence. Multiple sources may support one claim. The final column defines the inference supported by the cited evidence and identifies the next validation need. AP-1, activator protein 1; ATP, adenosine triphosphate; CSF1R, colony-stimulating factor 1 receptor; EAU, experimental autoimmune uveitis; HIF-1α, hypoxia-inducible factor 1 alpha; M1/M2, classically activated/alternatively activated macrophages; NAMPT, nicotinamide phosphoribosyltransferase; NIU, non-infectious uveitis; P2X7, P2X purinoceptor 7; RPE, retinal pigment epithelium.

A second pattern is the strong dependence of macrophage function on anatomical niche and disease stage. Resident microglia are positioned to sense retinal stress and shape local immune responses; retinal perivascular macrophages may regulate antigen presentation and leukocyte passage at venules; recruited monocyte-derived macrophages become more prominent during inflammatory amplification; and resident or recruited populations may later support debris clearance and repair. These roles can overlap, and current human datasets do not justify assigning a fixed pathogenic or reparative identity to any one population.

A third pattern is the separation between mechanistic evidence and therapeutic maturity. Tumor necrosis factor (TNF) blockade has established clinical efficacy in selected forms of NIU, yet the clinical benefit cannot be attributed specifically to macrophages. Janus kinase (JAK) and interleukin-6 (IL-6) pathway inhibitors have clinical signals but act across myeloid, lymphoid, vascular, and stromal compartments. By contrast, P2X7, chemokine receptors, individual inflammasome sensors, metabolic programs, and macrophage-state interventions are supported mainly by preclinical or exploratory human evidence.

### 2.2 Interpretation and reporting principles

Throughout this review, evidence provenance is treated as a set of complementary dimensions rather than a single hierarchy. Human intervention establishes clinical effects but may not identify the responsible cell type. Patient-derived studies establish that a state or association is present in the sampled compartment, whereas causal model perturbations test mechanism under defined experimental conditions. Associative model findings and cross-disease observations can support biological plausibility but should not be presented as direct NIU mechanisms. **Table 1** therefore pairs each major finding with the inference it supports and the principal unresolved question, rather than using the provenance abbreviations as a numerical score.

This article is a structured narrative review rather than a registered systematic review or meta-analysis. Quantitative pooling was not appropriate because diagnoses, sampling compartments, experimental models, interventions, and outcomes were heterogeneous. Interpretation at the claim level follows standardized uveitis nomenclature and distinguishes active immunization, adoptive transfer, and spontaneous models (27–30). Complete database-specific search strings, coverage dates, eligibility criteria, and evidence selection procedures are reported in **Supplementary Table S1** and are not repeated in the main text.

## 3 Cell origin and ocular myeloid niches

This section distinguishes ocular myeloid populations by developmental origin and anatomical niche before considering their functions in disease. The distinction is necessary because cells located in the neural retina, retinal vascular wall, vitreoretinal interface, and uveal tract differ in access to circulating mediators, local antigens, neural injury, and tissue repair signals.

### 3.1 Resident retinal microglia

Retinal microglia are long-lived macrophage-lineage cells derived from embryonic primitive progenitors rather than adult circulating monocytes, as established by fate-mapping studies (31, 32). Okunuki et al. used PLX5622, a CSF1R antagonist, and reported near-complete suppression of EAU after depletion of retinal resident myeloid cells (9). This intervention is not microglia specific: independent ocular studies show that CSF1R-directed strategies also affect choroidal and retinal perivascular macrophage compartments (19, 20). The PLX5622 experiment therefore supports a requirement for a CSF1R-dependent resident myeloid compartment in that model, but it does not prove that microglia alone initiate ocular inflammation. More selective deletion of MHC class II using Cre recombinase driven by the purinergic receptor P2Y12 (P2ry12) or transmembrane protein 119 (Tmem119) promoters reduced EAU severity without reproducing the near-complete protection observed after broad CSF1R antagonism (21). Although these studies were not designed as a head-to-head comparison, the lineage-restricted result supports antigen presentation mediated by microglial MHC II as an amplifier of local CD4+ T-cell responses, while leaving the initiating role of microglia unresolved.

Microglial activation should not be interpreted as a single pathogenic state. Interferon-responsive antigen presentation programs, chemokine release, purinergic sensing, phagocytosis, and homeostatic repair may coexist or appear sequentially. The M1/M2 vocabulary compresses these functions into an artificial binary and obscures tissue imprinting, metabolic state, and temporal plasticity. A more informative description specifies ontogeny, anatomical location, disease time point, activating signals, marker panel, and measured function. This distinction is particularly important in inflamed retina, where infiltrating macrophages may acquire microglia-like markers and resident cells may lose canonical homeostatic genes (33).

### 3.2 Retinal perivascular macrophages and vitreal hyalocytes

Retinal perivascular macrophages are anatomically and functionally distinct from choroidal and other uveal macrophages. They are associated mainly with retinal venules and occupy the interface between the endothelial compartment and the collagen IV/laminin-containing vascular sheath of the blood-retinal barrier; their precise position relative to this composite barrier remains unresolved (18, 20). These cells are enriched for MHC class II and pro-chemotactic programs, and experimental ablation reduces leukocyte recruitment in mouse ocular neuroinflammation. The available data therefore support a role in antigen presentation and vascular entry in experimental ocular inflammation, but they do not yet define the contribution of perivascular macrophages to EAU or human NIU. **Figure 1** depicts them as a distinct blood-retinal barrier population and includes them in the antigen presentation module.

Vitreal hyalocytes form a separate tissue-resident macrophage population on the vitreous side of the inner limiting membrane and at the vitreoretinal interface. Ontogeny studies identify them as long-lived yolk-sac-derived macrophages, and human transcriptional profiling distinguishes hyalocytes from blood-derived monocytes, conventional macrophages, and brain microglia (22–24). Their location permits interaction with the inner retinal surface, vitreous cytokines, and infiltrating leukocytes. However, their causal contribution to EAU and human uveitis has not been established. We therefore include hyalocytes as an anatomically relevant but evidence-limited population without assigning them a defined pathogenic function.

### 3.3 Uveal macrophages and choroidal dendritic cells

Uveal macrophages in the choroid, iris, and ciliary body occupy vascular and stromal environments that differ from the non-fenestrated retinal circulation. Choroidal macrophages lie adjacent to the retinal pigment epithelium (RPE) and fenestrated choriocapillaris, whereas iris and ciliary body macrophages encounter anterior uveal tissues and signals from aqueous humor. These populations may contribute differently to posterior, granulomatous, and anterior inflammatory phenotypes, but subtype-specific causal evidence in NIU remains sparse. Choroidal dendritic cells constitute a distinct antigen-presenting compartment and should not be subsumed under macrophages (19, 34). We therefore discuss each anatomical compartment separately rather than grouping these cells under the broad term border-associated macrophages.

### 3.4 Recruited monocyte-derived macrophages

Kinetic studies of leukocyte infiltration demonstrate dynamic entry of circulating monocytes during active EAU (15). Studies of oxidative and nitrosative injury link inflammatory cell accumulation to photoreceptor stress, but they do not assign each damaging mediator specifically to recruited macrophages (35–37). Recruitment also varies by model: C-C chemokine receptor type 2 (CCR2) is dispensable for efficient monocyte recruitment in induced EAU but is required in a spontaneous autoimmune uveitis model (38, 39). This difference argues against a universal recruitment mechanism and supports phenotype- and stage-specific evaluation of trafficking blockade.

### 3.5 The myeloid-glial-vascular network

Macrophage-lineage cells function within a reciprocal myeloid-glial-vascular network rather than as isolated effectors. Müller cell proteomics detected early inflammatory activation in autoimmune uveitis (40, 41), while retinal profiling identified MHC class II-positive antigen-presenting cells (42). Cluster of differentiation 44 (CD44) contributes to leukocyte trafficking across the blood-retinal barrier (43). Pharmacological β-catenin modulation preserved endothelial barrier integrity in a retinal vascular inflammation model outside the core EAU evidence set (44). The latter finding supports barrier protection as a therapeutic concept, but it does not establish a macrophage-specific mechanism in NIU.

Taken together, ocular myeloid populations should be defined by origin and location rather than pooled under a single label. The most direct EAU evidence concerns CSF1R-dependent resident myeloid cells, microglial MHC class II signaling, and recruited monocytes. The roles of retinal perivascular macrophages, vitreal hyalocytes, and individual uveal populations remain anatomically plausible but incompletely resolved in human disease.

## 4 Disease stage framework

The stage-based framework in **Figure 2** separates homeostasis, priming, neurovascular entry, inflammatory amplification, resolution or repair, and chronicity or relapse. At each stage, the figure identifies the dominant myeloid population and displays evidence provenance separately from therapeutic maturity. This organization is intended to prevent a model-derived mechanism, a patient association, and a clinically validated treatment from receiving the same interpretive weight.

### 4.1 Priming and loss of immune restraint

Peripheral tolerance and systemic immune activation precede ocular inflammation in several models. Autoimmune regulator (AIRE)-dependent thymic tolerance restricts ocular autoimmunity in a transgenic model expressing an autoreactive T-cell receptor (TCR) (45), whereas microbiota-dependent activation can trigger autoreactive T cells in spontaneous ocular autoimmunity (46). Short-chain fatty acids can also alter lymphocyte trafficking in immune-mediated uveitis (47). These studies identify systemic inputs that may condition circulating monocytes and resident microglia, but they do not establish macrophages as the initiating cell in every NIU phenotype.

Within the retina, microglia can respond to extracellular adenosine triphosphate (ATP), RPE dysfunction, neuronal stress, and low-level cytokine signals, although the transition from surveillance to pathogenic activation remains stage dependent. One translational study associated poor sleep quality with more severe inflammation in patients and, in a separate EAU experiment, showed that chronic sleep deprivation increased signal transducer and activator of transcription 1 (STAT1) phosphorylation, interferon-stimulated gene 15 (ISG15) expression, macrophage activation, and disease severity (48). The patient finding is observational, whereas the mechanistic perturbation was performed in the model; the two components therefore support different levels of inference.

### 4.2 Neurovascular entry and local restimulation

Neurovascular entry occurs at specialized vascular sites where endothelium, retinal perivascular macrophages, other antigen-presenting cells, chemokines, monocytes, and autoreactive T cells interact. Retinal perivascular macrophages are associated with postcapillary venules, express MHC class II and pro-chemotactic programs, and regulate leukocyte recruitment in mouse ocular neuroinflammation (18, 20). These findings support gatekeeping and antigen presentation functions at the blood-retinal barrier but do not establish that these cells are required in EAU. The role of vitreal hyalocytes in leukocyte entry or local restimulation remains unknown. In EAU, interleukin-27 (IL-27) deficiency augments T helper 1 (Th1) and T helper 17 (Th17) responses (49), whereas interleukin-17A (IL-17A) can limit Th17 pathogenicity through negative feedback mediated by interleukin-24 (IL-24) (50). Aging can also reduce Th17 pathogenicity in the model (51). Human acute anterior uveitis samples implicate interleukin-22 (IL-22), granulocyte-macrophage colony-stimulating factor (GM-CSF), and interleukin-17F (IL-17F), but soluble cytokine measurements alone cannot identify the producing ocular cell (52).

C-X-C chemokine receptor type 3 (CXCR3) antagonism with AMG487 altered immune cell programs and reduced EAU severity (53). A separate study combining single-cell analysis with experimental perturbation identified activator protein 1 (AP-1) as a candidate target (54), whereas studies of histone deacetylase (HDAC), cyclin-dependent kinase 6 (CDK6), inhibitor of DNA binding 2 (ID2), and Ras-related C3 botulinum toxin substrate 1 (Rac1) primarily resolved pathogenic T-cell programs (55, 56). These reports address different mechanisms and dominant cell compartments; they should not be combined as evidence for a macrophage-specific therapy.

### 4.3 Peak amplification and tissue injury

At peak EAU, inflammatory cell accumulation coincides with mitochondrial oxidative stress in photoreceptors (35). Studies of free radicals and nitric oxide further link oxidative and peroxynitrite/Fas-associated pathways to photoreceptor apoptosis (36, 37). Gasdermin-mediated inflammatory cell death is well defined in the broader pyroptosis literature (57), but its sensor-specific and cell-specific contribution to NIU still requires direct testing. Lipocalin-2 is increased in EAU retina, although the available study does not establish it as a causal macrophage effector (58).

P2X7 regulates antigen-presenting cell function in EAU (59), and macrophage-specific P2X7 expression controls inflammasome-, complement-, and phagocytosis-related programs in autoimmune uveitis (60). P2X7 signaling in effector T cells represents a separate, non-myeloid mechanism (61). Inflammasome effects also depend on the sensor and experimental context. NOD-like receptor family pyrin domain-containing 3 (NLRP3) contributes to chronic EAU in one study (62), whereas another study found that NLRP3 restrained pyroptosis mediated by absent in melanoma 2 (AIM2) through salt-inducible kinase 1 (SIK1)/sterol regulatory element-binding transcription factor 1 (SREBF1) signaling (63). NOD-like receptor family pyrin domain-containing 12 (NLRP12), in turn, suppresses retinal autoimmunity (64). A general inflammasome synthesis (65) and a 2024 review of retinal disease (66) provide useful background but are not direct NIU evidence. Therapeutic studies should therefore identify the sensor, cell subset, disease stage, and downstream effector being targeted.

### 4.4 Resolution and incomplete recovery

Resolution requires lower inflammatory output together with preserved efferocytosis, debris clearance, lipid handling, and barrier support. Interleukin-33 (IL-33) attenuated EAU and promoted features associated with alternatively activated macrophages (67). Modulation of the adrenomedullin–receptor activity-modifying protein 2 (RAMP2) axis reduced retinal T-cell and macrophage infiltration while increasing regulatory T cell (Treg) and M2-like populations in EAU (68). In a retinal injury model rather than uveitis, a recruited macrophage subset promoted axonal regrowth (69). This cross-disease finding supports the possibility of macrophage-mediated repair but cannot be treated as direct evidence of NIU resolution.

Other interventions also affect macrophage-associated state or metabolic markers. Baicalin reduced EAU severity and altered hypoxia-inducible factor 1 alpha (HIF-1α), mitochondrial potential, reactive oxygen species (ROS), inducible nitric oxide synthase (iNOS), and arginase 1 (ARG1) measures (70). Recombination signal binding protein for immunoglobulin kappa J region (RBPJ) knockdown favored an M2-like program through mitochondrial ROS and signaling involving Notch receptor 1 (Notch1), Jagged canonical Notch ligand 1 (Jagged1), and hairy and enhancer of split 1 (HES1) (71), while purified corticotropin gel showed activity in chronic EAU (72). These studies support separate preclinical mechanisms; they do not establish a common reparative macrophage state or clinical efficacy.

### 4.5 Chronicity, relapse, and innate memory

Trained immunity refers to durable functional reprogramming of innate cells and involves epigenetic and metabolic pathways, including glycolysis regulated by mechanistic target of rapamycin (mTOR) and HIF-1α, as well as the mevalonate pathway (73–75). Innate immune memory in microglia has also been demonstrated in other central nervous system settings (76). In 2026, a study of ankylosing spondylitis-associated acute anterior uveitis identified cluster of differentiation 14-positive and intercellular adhesion molecule 1-high (CD14+ICAM1-high) monocytes with trained-immunity-like transcriptional, functional, epigenetic, and metabolic features. Single-cell analysis of aqueous humor identified related macrophage states, and pseudotime analysis suggested a transition from circulating monocytes toward ocular macrophages (77). This study provides direct patient-linked evidence for a trained-immunity-like myeloid state in a defined NIU phenotype, but the proposed transition remains a computational inference rather than lineage tracing. Chronic EAU inflammasome activity and time-dependent microglial transcription provide complementary model evidence (62, 78). Whether these states persist through remission or predict relapse will require longitudinal paired blood and ocular sampling.

Across the disease course, the same myeloid population may perform different functions as local signals change. Evidence is strongest for model-dependent roles in entry and inflammatory amplification, whereas resolution, durable reprogramming, and relapse prediction remain less well established. Stage should therefore be specified whenever a macrophage mechanism or therapeutic target is discussed.

## 5 Evidence from experimental models and patients

Experimental models and patient samples provide different but complementary forms of evidence. The following subsections define what each source can establish, where cellular attribution remains uncertain, and which study designs are needed to connect model-derived mechanisms with clinically measurable human states.

### 5.1 What EAU establishes—and what it cannot

EAU permits controlled disease timing, retinal tissue analysis, and causal perturbation, but its variants answer different questions. Active immunization emphasizes peripheral priming with retinal antigen and adjuvant, whereas adoptive transfer isolates entry of effector cells and local restimulation (8). Spontaneous models test endogenous tolerance and may reveal dependencies that are absent from induced disease, as illustrated by the different requirements for CCR2 (38, 39). Kinetic studies of leukocyte infiltration demonstrate dynamic myeloid influx (15), while behavioral conditioning experiments illustrate systemic modulation of model activity (79). No single model reproduces the full range of anterior, pediatric, granulomatous, relapsing, or treatment-experienced human NIU (6).

Recent intervention studies have improved mechanistic resolution, but each result should be mapped to its specific target and dominant cellular compartment. Ripasudil reduced acute EAU (80); baicalin and RBPJ knockdown altered macrophage-associated metabolic or state markers (70, 71); AP-1 targeting and CXCR3 antagonism combined single-cell discovery with model intervention (53, 54); adrenomedullin–RAMP2 modulation affected vascular, T-cell, and macrophage compartments (68); and chronic sleep deprivation increased macrophage pathogenicity (48). Because these interventions act on multiple cell types, a lower retinal cluster of differentiation 11b-positive (CD11b+) cell count or altered M1/M2 ratio alone does not demonstrate macrophage-specific action.

### 5.2 Patient ocular samples: what each compartment can establish

Aqueous and vitreous samples allow measurement of cytokines and chemokines, but bulk soluble concentrations usually do not identify the producing cell (30). Vitreous IL-6 correlated with widefield optical coherence tomography (OCT) particle findings in posterior uveitis (81), whereas interleukin-10 (IL-10) and the IL-6/IL-10 ratio showed predictive value for treatment response in a separate cohort (82). These observations support clinically accessible inflammatory phenotyping. However, mixed diagnoses, treatment exposure, sampling time, and the absence of cell attribution limit macrophage-specific interpretation.

Single-cell studies partly address this limitation. Single-cell RNA sequencing (scRNA-seq) of immune cells from aqueous humor obtained from patients with uveitis identified macrophages as candidate sources of conserved C-C motif chemokine ligand 2 (CCL2) and C-X-C motif chemokine ligand 10 (CXCL10) gradients and mapped ligand-receptor networks consistent with recruitment of additional inflammatory cells (25). A subsequent paired ocular fluid and blood study of 23 patients showed disease-associated differences in immune cell composition: HLA-B27-associated acute anterior uveitis was enriched for myeloid cells and innate immune programs, whereas chronic uveitis showed stronger adaptive immune populations and transcriptional programs (26). These studies provide direct, cell-resolved human observations. Nevertheless, transcript abundance does not prove protein secretion, aqueous cells do not represent every retinal or choroidal niche, and cross-sectional sampling cannot establish lineage origin or temporal causality.

The sampling compartment determines the claim that can be supported. Aqueous humor can be obtained during clinically indicated anterior chamber procedures, but a concentration change may reflect production by the iris or ciliary body, diffusion from posterior disease, barrier leakage, or altered clearance. Vitreous samples are closer to retinal and choroidal inflammation and can be paired with OCT, angiography, or vitritis grading, but they are usually collected only during surgery or diagnostic vitrectomy. Blood permits serial assessment of systemic immune states but cannot establish ocular residence. Retinal, choroidal, or iris tissue provides spatial context and cell identity, although available specimens are uncommon, surgically selected, and often obtained after substantial immunosuppression. A macrophage-related claim is therefore most persuasive when cellular phenotype, ocular location, functional readout, and clinical activity are measured in the same patient.

Several human studies define immune circuits that interact with, but do not directly establish, macrophage mechanisms. Patient samples from acute anterior uveitis implicated IL-22, GM-CSF, and IL-17F (52). In Behcet’s uveitis, one study described an amplification loop involving neutrophil extracellular traps (NETs), CD44, and IL-17A (83), and another associated circulating unconventional neutrophils with sex-specific immunological outcomes (84). Single-cell profiling identified abnormal naive CD4+ T-cell differentiation (85), while a comprehensive review summarizes the wider disease context (86). These findings should be cited for the pathways they directly examine rather than presented as macrophage-specific evidence.

### 5.3 Patient-derived functional and longitudinal evidence

Experiments using patient-derived monocytes from patients with Behcet’s uveitis and Vogt–Koyanagi–Harada syndrome showed that adalimumab altered inflammatory macrophage programs and reduced support for Th1 and Th17 responses (87). Because the cells were differentiated ex vivo, they are not equivalent to microglia or macrophages conditioned within an inflamed eye. Nicotinamide phosphoribosyltransferase (NAMPT) research combined EAU perturbation with single-cell data from peripheral blood in Vogt–Koyanagi–Harada disease and identified a conserved program involving NAMPT and HIF-1α primarily in CD4+ T cells (88). This finding supports cross-compartment pathway conservation but does not establish retinal macrophages as the dominant human target.

Patient samples become more informative when they distinguish active disease, treatment response, quiescence, and relapse. A cross-sectional cytokine elevation may accompany tissue damage without causing it, whereas a baseline signature that predicts a prespecified clinical response is closer to a clinically useful biomarker. Observations involving IL-10 and the IL-6/IL-10 ratio illustrate this progression, although external validation, assay harmonization, phenotype-specific thresholds, and adjustment for current therapy remain necessary. Vitreous IL-6 measurements linked to imaging similarly connect a molecular measurement with an ocular phenotype but do not show that serial IL-6 monitoring improves clinical decisions. Future studies should prespecify sampling in relation to corticosteroid exposure, use standardized activity definitions, report anatomical subtype and systemic association, and retain paired blood and ocular aliquots for orthogonal validation (29, 30, 81, 82).

Evidence should also be classified by its intended clinical use. Diagnostic evidence determines whether a signature distinguishes inflammatory disease from non-inflammatory or infectious mimics. Prognostic evidence estimates the risk of relapse, edema, vasculitis, or visual loss. Predictive evidence identifies patients who are more likely to benefit from a particular intervention, and pharmacodynamic evidence shows whether that intervention alters its intended pathway. These purposes are not interchangeable. A marker associated with active vitritis may be clinically useful without proving causal macrophage biology, while a target engagement marker may support mechanism even if it lacks diagnostic specificity.

### 5.4 Evidence gaps in human tissue

Direct sampling of inflamed retina and choroid is uncommon, and enucleation or surgical specimens represent selected, often late-stage disease. Aqueous humor is more accessible but may not reflect posterior retinal niches. Peripheral blood is useful for systemic signatures but cannot establish ocular residence or function. Single-cell dissociation may underrepresent fragile or adherent cells and removes spatial relationships; during inflammation, microglia and monocyte-derived macrophages may also converge transcriptionally. The main unmet need is therefore a longitudinal, spatially resolved human atlas linking ocular compartment, treatment exposure, disease activity, imaging, and visual outcomes to validated myeloid states.

A practical pathway for human evidence should be viewed as a sequence of complementary tests rather than an ordinal hierarchy. Paired ocular fluid and blood profiling addresses compartment concordance; cell-resolved or spatial confirmation tests cellular attribution; and target engagement linked to intervention determines whether treatment changes the pathway. Archived trial samples may support retrospective discovery of cytokine or monocyte-state predictors, but each signature requires prospective validation in a prespecified phenotype. Multicenter biobanking will be particularly important for less common but mechanistically coherent groups, including birdshot chorioretinopathy, Behcet’s retinal vasculitis, and treatment-naïve JIA-associated uveitis.

The central evidentiary conclusion is that human ocular studies now provide cell-resolved observations, but they still rarely establish lineage, temporal sequence, or causal function. Progress will require longitudinal sampling across activity states, spatial or protein-level confirmation, and prospective validation of biomarkers within clearly defined NIU phenotypes.

## 6 Therapeutic targets and clinical maturity

Current NIU treatment includes corticosteroids, conventional immunomodulators, biologics, and targeted small molecules (89). This section focuses on pathways with a plausible macrophage contribution while recognizing that most approved or clinically tested agents act across several immune, vascular, and stromal compartments. Clinical efficacy is therefore evaluated separately from evidence of macrophage-specific mechanism.

### 6.1 Clinically anchored cytokine and signal-transduction pathways

TNF neutralization modifies immune function in EAU (90), but clinical efficacy is established by separate human trials. VISUAL I randomized 217 adults with active intermediate, posterior, or panuveitis and showed that adalimumab prolonged the median time to treatment failure from 13 to 24 weeks (91). VISUAL II randomized 226 adults with corticosteroid-controlled inactive disease and reduced the risk of treatment failure (92). These trials validate TNF as a therapeutic pathway, but their endpoints and drug effects span myeloid, lymphoid, vascular, and stromal compartments and do not establish an mechanism specific to ocular macrophages.

In SYCAMORE, 90 patients with JIA-associated uveitis receiving methotrexate were randomized to adalimumab or placebo; treatment failure occurred in 27% and 60%, respectively (93). VISUAL III provided open-label follow-up (94), and a 2024 cohort reported maintained disease control after switching to the SB5 biosimilar, although device tolerability affected treatment persistence (95). A separate experiment using patient-derived macrophages supports a plausible effect of adalimumab on myeloid state (87), but it does not prove that macrophage reprogramming mediates the clinical benefit observed in the trials.

STOP-Uveitis was a randomized, open-label dose study of 37 patients receiving intravenous tocilizumab; at month 6, visual acuity, central macular thickness, and vitreous haze outcomes showed improvement signals (96). SATURN randomized 58 patients to sarilumab or placebo. The central reader vitreous haze endpoint was not significant, although investigator-graded haze and visual acuity outcomes favored sarilumab (97). These trials provide a clinical signal for IL-6 pathway inhibition, but their designs and outcomes are not equivalent and neither validates a macrophage-specific mechanism.

Local IL-6 inhibition has also entered late-stage clinical development. DOVETAIL was an open-label, nonrandomized, multiple-ascending-dose phase 1 study of intravitreal vamikibart, an anti-IL-6 monoclonal antibody, in uveitic macular edema secondary to NIU. The peer-reviewed report supports preliminary safety and tolerability and describes possible anatomical and visual effects, but its design and sample size do not establish comparative efficacy (98). The sponsor later reported completion of the phase 3 MEERKAT and SANDCAT programs, with statistically significant vision improvement in MEERKAT and nominal significance in SANDCAT (99). Because full peer-reviewed phase 3 results and regulatory review were unavailable at the search cutoff, these findings are presented as a development update rather than evidence of approval or definitive efficacy.

HUMBOLDT randomized 74 patients with active NIU and reported treatment failure by week 24 in 37.5% of the filgotinib group and 67.6% of the placebo group; early trial termination limited exposure and the precision of safety estimates (100). Retrospective experience with JAK inhibitors (101), tofacitinib case reports (102), and upadacitinib data from EAU (103) provide less controlled and mechanistically distinct forms of support. Because JAK inhibition affects lymphocytes, myeloid cells, endothelium, and stromal cells, clinical response should not be equated with macrophage-specific target validation.

An open-label pilot study of gevokizumab provides an early clinical signal for interleukin-1 beta (IL-1β) blockade in resistant Behcet’s uveitis (104). In EAU, NLRP3, AIM2, and NLRP12 have non-equivalent and sometimes opposing functions (62–64). Abatacept has been used in severe JIA-associated uveitis refractory to anti-TNF therapy (105), but this experience is an indirect test of antigen-presenting cell and T-cell reciprocity rather than selective ocular myeloid targeting.

### 6.2 Preclinical myeloid targets

P2X7 is a macrophage-proximal preclinical target supported by studies of antigen-presenting cells, macrophage-specific signaling, and effector T cells in EAU (59–61). CCR2 dependence differs between induced and spontaneous disease (38, 39), whereas CXCR3 antagonism has support from model intervention but no NIU trial (53). Other targets require individual interpretation: TANK-binding kinase 1 (TBK1)/nuclear factor kappa B (NF-κB) (106), AP-1 (54), HIF-1α and NAMPT (88, 107), RBPJ/Notch and state modulation directed at HIF-1α (70, 71), and Rho-associated coiled-coil-containing protein kinase (ROCK) (80). These pathways share several translational liabilities, including pleiotropy, uncertain ocular exposure, and possible suppression of protective myeloid functions.

Therapeutic maturity is reported independently of evidence provenance. Clinically established or trial-anchored pathways include TNF, IL-6, and Janus kinase–signal transducer and activator of transcription (JAK–STAT) (91, 92, 96, 97, 100). Macrophage-proximal inflammatory or trafficking targets, including P2X7, individual inflammasome sensors, CCR2, and CXCR3, remain preclinical (38, 39, 53, 59, 60, 62–64). State reprogramming and repair-oriented targets, including AP-1, HIF-1α, NAMPT, RBPJ/Notch, ROCK, and adrenomedullin–RAMP2, remain hypothesis-generating or preclinical (54, 68, 70, 71, 80, 88, 107). These categories describe development readiness and should not be interpreted as a ranking of mechanistic validity.

**Table 2** compares each target by implicated cell subset, supporting evidence, clinical maturity, and major translational limitation. The development strategy should be matched to the target. Trafficking inhibitors require direct evidence of reduced ocular entry; P2X7 or inflammasome inhibitors require sensor- and compartment-specific pharmacodynamic markers; metabolic or transcriptional modulators require confirmation of the intended state change without retinal toxicity or loss of phagocytic repair; and barrier-directed strategies require measures of vascular leakage and leukocyte passage.

**Table 2:** Macrophage-relevant therapeutic targets, implicated cell subsets, supporting evidence, clinical maturity, and principal translational limitations.

Target	Cell subset	Evidence source	Clinical maturity	Major translational limitation
TNF	Activated monocyte-derived macrophages; microglia; other TNF-producing cells	EAU neutralization; VISUAL I/II/III; JIA trial; patient macrophage study (87, 90–94)	Established pathway; approved adalimumab for selected NIU	Systemic immunosuppression, infection risk, and absent ocular myeloid selectivity.
IL-6/IL-6R	Inflammatory macrophages, stromal cells, T-cell circuit	Ocular fluid cohorts; STOP-Uveitis; SATURN (81, 82, 96, 97)	Clinical signal and late-stage development; peer-reviewed phase 3 evidence pending	Heterogeneous trials, non-myeloid pathway effects, and incomplete peer-reviewed phase 3 reporting.
JAK–STAT	Macrophages, T cells, and stromal cells	HUMBOLDT phase 2; retrospective JAK inhibitor series; EAU single-cell studies (100, 101, 103)	Phase 2 signal; not macrophage selective	Class safety concerns, premature trial termination, and unresolved ocular target engagement.
IL-1/inflammasome	Macrophages with active inflammasome signaling and microglia	EAU NLRP3/AIM2/NLRP12 studies; Behçet pilot (62–64, 104)	Early clinical/preclinical; context dependent	Context-dependent inflammasome biology and limited controlled human efficacy evidence.
P2X7	Microglia, recruited macrophages, APCs	Genetic and pharmacological EAU studies (59–61)	Preclinical with strong target-proximal evidence	Broad cellular expression, liabilities related to host defense, and no human ocular target engagement data.
CCR2/CXCR3 trafficking	Circulating monocytes and recruited macrophages	Induced versus spontaneous EAU; AMG487 study (38, 39, 53)	Preclinical; model and stage dependent	Recruitment redundancy and strong dependence on model, phenotype, and intervention timing.
TBK1/NF-κB/AP-1	Activated ocular myeloid cells and interacting lymphocytes	EAU pathway inhibition and scRNA-seq (54, 106)	Preclinical	Ubiquitous signaling, off-target toxicity, and inadequate cell-selective delivery.
HIF-1α/NAMPT/lactylation	Metabolically activated microglia/macrophages; CD4+ T cells	EAU perturbation, single-cell data, VKH blood validation (70, 88, 107, 109)	Preclinical/biomarker discovery stage	Pathway pleiotropy, uncertain ocular pharmacology, and no validated selection biomarker.
ROCK	Microglia/macrophages and T cells	Acute EAU with ripasudil (80)	Preclinical repurposing candidate	Multicellular effects, evidence from an acute model, and uncertain exposure in posterior tissue.
RBPJ–Notch	Macrophages undergoing state transition	Knockdown and mitochondrial ROS experiments in EAU (71)	Preclinical	Pleiotropic developmental signaling and lack of a clinically feasible delivery strategy.
Adrenomedullin–RAMP2	M2-like macrophages, Tregs, vascular cells	Knockout and recombinant peptide EAU experiments (68)	Preclinical; repair-oriented	Peptide stability, systemic vascular effects, and no direct human uveitis evidence.
Barrier protection/tissue resilience	Endothelium, RPE, perivascular macrophages	CD44 trafficking and β-catenin barrier studies (43, 44)	Preclinical adjunctive strategy	Indirect immune control and uncertain efficacy after established inflammatory injury.

AIM2, absent in melanoma 2; AP-1, activator protein 1; APC, antigen-presenting cell; CCR2, C-C chemokine receptor type 2; CXCR3, C-X-C chemokine receptor type 3; EAU, experimental autoimmune uveitis; HIF-1α, hypoxia-inducible factor 1 alpha; IL, interleukin; IL-6R, interleukin-6 receptor; JAK–STAT, Janus kinase–signal transducer and activator of transcription; JIA, juvenile idiopathic arthritis; M2, alternatively activated macrophage; NAMPT, nicotinamide phosphoribosyltransferase; NF-κB, nuclear factor kappa B; NIU, non-infectious uveitis; NLRP3/NLRP12, NOD-like receptor family pyrin domain-containing 3/12; P2X7, P2X purinoceptor 7; RAMP2, receptor activity-modifying protein 2; RBPJ, recombination signal binding protein for immunoglobulin kappa J region; ROCK, Rho-associated coiled-coil-containing protein kinase; ROS, reactive oxygen species; RPE, retinal pigment epithelium; scRNA-seq, single-cell RNA sequencing; TBK1, TANK-binding kinase 1; TNF, tumor necrosis factor; Treg, regulatory T cell; VKH, Vogt–Koyanagi–Harada.

### 6.3 Translational barriers

Disease heterogeneity is the first translational barrier. NIU comprises multiple syndromes rather than a single mechanism: an intervention developed in posterior EAU may be less relevant to HLA-B27 anterior uveitis, JIA-associated chronic anterior disease, granulomatous sarcoid uveitis, or neutrophil-rich Behcet’s retinal vasculitis. Anatomical classification alone is also insufficient because patients with the same anatomical diagnosis may differ in systemic association, disease duration, treatment exposure, vascular injury, and dominant immune circuit. Broad enrollment without biomarker stratification may therefore obscure an effect confined to a mechanistically coherent subgroup.

Cell identity and disease timing form a second barrier. During inflammation, recruited macrophages may acquire microglia-like transcriptional features, while resident microglia may lose homeostatic markers; a cluster label or isolated surface marker therefore does not establish ontogeny. The optimal intervention may also change over time. Blocking CCR2-dependent entry could be useful during vascular recruitment but harmful when infiltrating cells are clearing debris. Inflammasome inhibition may reduce acute injury but have little effect on fixed edema or ischemia, whereas repair-oriented reprogramming may be premature before inflammation is controlled. Cross-sectional sampling obscures these transitions and encourages interpretation of a dynamic process as a fixed cell state.

Model design is a third barrier. Many EAU studies begin treatment before or soon after immunization, use one strain and antigen, and end at peak clinical score. Patients usually present with established disease, concurrent corticosteroid exposure, previous immunomodulator failure, and a continuing risk of relapse or structural damage. A candidate treatment should therefore be tested with delayed dosing in more than one induction system, on a clinically relevant treatment background, and in both sexes. Outcomes should extend beyond fundus score to OCT, angiography, electroretinography or visual behavior, relapse after withdrawal, and histological evidence of repair.

Tissue access and biomarker validation form a fourth barrier. Aqueous cytokines, vitreous particles on OCT, peripheral monocyte states, circulating proteins, and extracellular vesicle signals are feasible candidates, but none currently defines a validated macrophage-enriched treatment population. Each assay must be analytically stable across centers, collected at a specified interval from local or systemic therapy, and prospectively linked to a clinical decision. Blood biomarkers are strongly influenced by systemic inflammation and drug exposure, whereas ocular biomarkers are closer to the target organ but require invasive sampling and may not represent all retinal niches.

Pharmacology and safety form a fifth barrier. The blood-retinal barrier may limit ocular exposure, whereas local delivery can produce steep retinal concentration gradients, burden associated with repeated injections, endophthalmitis risk, and off-target retinal toxicity. Systemic macrophage inhibition can impair antimicrobial defense, wound healing, lipid handling, and tissue homeostasis. Permanent depletion is particularly difficult to justify because microglia and macrophages contribute to debris clearance, vascular stabilization, and resolution. Reversible state modulation, short exposure during a defined disease stage, or ligand-directed delivery may provide a wider therapeutic window, but each strategy requires ocular pharmacokinetic and pharmacodynamic confirmation.

Trial design is the final barrier. Composite failure endpoints are efficient but may combine biologically distinct events, while vitreous haze grading can differ between central readers and investigators, as illustrated by SATURN (97). Visual acuity may remain stable despite active angiographic leakage and may also be limited by irreversible damage after inflammation has resolved. Early trials designed to test mechanism should therefore combine a prespecified inflammatory endpoint with OCT or angiography, steroid-sparing exposure, patient-reported visual function, and a target engagement measure. Randomization should be stratified by anatomical phenotype and baseline therapy, with relapse and safety followed beyond the initial response period.

### 6.4 A development pathway guided by disease stage

A macrophage-relevant program should enter clinical testing only after five questions have been addressed: which NIU phenotype is intended; which ocular compartment and myeloid subset express the target; at which disease stage the intervention is expected to work; how ocular target engagement will be measured; and which reparative or systemic functions might be compromised. Discovery cohorts can nominate a cell state, but advancement requires orthogonal protein or spatial confirmation. Preclinical efficacy should be demonstrated with delayed treatment and clinically interpretable outcomes. An early human study should then establish ocular exposure or a credible systemic surrogate before a larger efficacy trial.

The corresponding trial population should be enriched by mechanism while remaining clinically recognizable. Candidate entry variables include an IL-6/IL-10 pattern, a prespecified chemokine or monocyte-state module, inflammatory particles or hyperreflective foci on OCT, active vascular leakage, or a disease-stage signature. The biomarker does not need to be unique to macrophages, but it must predict target presence or pharmacological response and be prespecified before confirmatory testing. Serial aqueous sampling may be appropriate in selected proof of mechanism studies when coordinated with clinically indicated ocular procedures; otherwise, paired blood, imaging, and steroid-sparing endpoints offer a less invasive design.

Overall, clinical maturity is highest for broad cytokine pathways and lowest for macrophage-selective trafficking, inflammasome, metabolic, and repair strategies. Advancement should depend on a defined patient phenotype, a stage-specific mechanism, measurable ocular target engagement, and evidence that protective myeloid functions are preserved.

## 7 Discussion and future directions

The major research priority is to convert descriptive myeloid states into testable, clinically linked mechanisms. This requires study designs that connect cell identity, anatomical compartment, disease stage, treatment exposure, and patient outcome rather than analyzing each element in isolation.

The next phase of research should connect cell identity and disease stage with longitudinal human outcomes. Patient cohorts should use harmonized anatomical and etiological classifications, document current and cumulative immunosuppression, and sample active disease, early response, quiescence, and relapse. Aqueous humor, blood, OCT, angiography, and clinical outcomes can be integrated even when retinal biopsy is not feasible. Single-cell and spatial analyses should include orthogonal protein or imaging validation and should not equate transcriptional clusters with fixed cell types. Paired ocular and peripheral samples will be particularly important for determining whether a blood signature reflects, or merely accompanies, ocular inflammation.

Preclinical studies should incorporate delayed treatment, clinically relevant corticosteroid backgrounds, both sexes, multiple EAU models, and longer follow-up for relapse, repair, and visual function. Conditional genetics or fate mapping should distinguish resident microglia from monocyte-derived macrophages. Drug development should favor reversible state modulation or targeted ocular delivery over permanent depletion. A target should advance only when its dominant cell subset, stage-specific window, ocular target engagement, and safety consequences can be demonstrated.

In summary, a useful trial architecture will be biomarker enriched rather than macrophage exclusive. TNF or JAK inhibition may be most relevant when cytokine and systemic immune signatures predominate; recruitment blockade may fit an early vascular entry phenotype; P2X7 or inflammasome modulation may require evidence of ATP- or IL-1-associated activity; and repair-oriented approaches may be appropriate only after inflammatory control. Matching the intervention to a measurable mechanism and disease stage should make both positive and negative trials more interpretable.

## 8 Conclusion

Macrophages are not a uniform downstream effector population in NIU. Resident microglia, retinal perivascular macrophages, vitreal hyalocytes, uveal macrophages, and recruited monocyte-derived macrophages occupy distinct anatomical niches and contribute differently across priming, entry, amplification, tissue injury, resolution, and relapse. The supporting evidence should be interpreted by provenance rather than collapsed into a single ordinal grade. Uveitis models provide several causal perturbations, human studies establish selected inflammatory states and treatment effects, and cross-disease findings provide context without proving NIU mechanisms. The 2026 study of ankylosing spondylitis-associated acute anterior uveitis adds patient-linked evidence for trained-immunity-like monocytes and related macrophage states in aqueous humor, while leaving lineage transition and relapse causality unresolved. TNF blockade is clinically established, and JAK inhibition has randomized phase 2 support; most macrophage-proximal targets remain preclinical. Translation will depend on matching a defined cell state and disease stage to a measurable human phenotype while preserving the myeloid functions required for retinal recovery.

## Glossary

AIM2: absent in melanoma 2
AI: artificial intelligence
AIRE: autoimmune regulator
AP-1: activator protein 1
APC: antigen-presenting cell
ARG1: arginase 1
ATP: adenosine triphosphate
BRB: blood-retinal barrier
C3: complement component 3
C5a: complement component 5a
CCL2: C-C motif chemokine ligand 2
CCR2: C-C chemokine receptor type 2
CD: cluster of differentiation
CDK6: cyclin-dependent kinase 6
CSF1R: colony-stimulating factor 1 receptor
CTLA-4-Ig: cytotoxic T-lymphocyte-associated protein 4 immunoglobulin
CXCL10: C-X-C motif chemokine ligand 10
CXCR3: C-X-C chemokine receptor type 3
EAU: experimental autoimmune uveitis
GM-CSF: granulocyte-macrophage colony-stimulating factor
H-D: human-derived evidence
H-I: human intervention
HDAC: histone deacetylase
HIF-1α: hypoxia-inducible factor 1 alpha
HLA-B27: human leukocyte antigen B27
HES1: hairy and enhancer of split 1
ICAM1: intercellular adhesion molecule 1
ID2: inhibitor of DNA binding 2
IL: interleukin
IL-6R: interleukin-6 receptor
iNOS: inducible nitric oxide synthase
ISG15: interferon-stimulated gene 15
JAK: Janus kinase
JAK–STAT: Janus kinase–signal transducer and activator of transcription
JIA: juvenile idiopathic arthritis
M1/M2: classically activated/alternatively activated macrophages
M-A: associative evidence from uveitis models
M-C: causal evidence from uveitis models
MHC II: major histocompatibility complex class II
mTOR: mechanistic target of rapamycin
NAMPT: nicotinamide phosphoribosyltransferase
NETs: neutrophil extracellular traps
NF-κB: nuclear factor kappa B
NIU: non-infectious uveitis
NLRP3: NOD-like receptor family pyrin domain-containing 3
NLRP12: NOD-like receptor family pyrin domain-containing 12
OCT: optical coherence tomography
P2X7: P2X purinoceptor 7
pvMac: retinal perivascular macrophage
RAMP2: receptor activity-modifying protein 2
RBPJ: recombination signal binding protein for immunoglobulin kappa J region
ROCK: Rho-associated coiled-coil-containing protein kinase
RNS: reactive nitrogen species
ROS: reactive oxygen species
RPE: retinal pigment epithelium
scRNA-seq: single-cell RNA sequencing
SIK1: salt-inducible kinase 1
SREBF1: sterol regulatory element-binding transcription factor 1
STAT1: signal transducer and activator of transcription 1
TBK1: TANK-binding kinase 1
TCR: T-cell receptor
Th: T helper
TNF: tumor necrosis factor
Treg: regulatory T cell
VKH: Vogt–Koyanagi–Harada
X: cross-disease context
