# Supplementary material for: Macrophages in non-infectious uveitis: cell origin, disease stage, evidence provenance, and therapeutic maturity
Source: Front Immunol. 2026 Sep 28;17:1958864. doi: 10.3389/fimmu.2026.1958864 (PMC13656210; doi:10.3389/fimmu.2026.1958864)
Supplement: Supplementary file 1 [file Table1.docx]

**Supplementary Table S1**

**Literature Search Strategy and Evidence Selection Procedures**

This table reports the database-specific search strategy, eligibility criteria, evidence-provenance framework, and synthesis procedures used for the structured narrative review.

| **Component** | **Database or criterion** | **Search specification or decision rule** |
| --- | --- | --- |
| **Review design** | Review type | Structured narrative review of macrophage-lineage biology in non-infectious uveitis (NIU). The synthesis was organized by cell origin, disease stage, evidence provenance, and therapeutic maturity. |
| **Search coverage** | Databases | PubMed/MEDLINE, Embase, Scopus, and Web of Science Core Collection. |
| **Search coverage** | Date range and last search | Database inception through 15 August 2026. The final targeted DOI, PMID, and title checks were completed on the same date. |
| **Search coverage** | Language | English-language records. |
| **Core concepts** | Ocular inflammation block | uveitis; non-infectious or noninfectious uveitis; autoimmune uveitis; experimental autoimmune uveitis; experimental autoimmune uveoretinitis; uveoretinitis; ocular inflammation. |
| **Core concepts** | Myeloid-cell block | macrophage; microglia; monocyte; myeloid cell; hyalocyte; perivascular macrophage; choroidal macrophage; uveal macrophage; iris macrophage; ciliary body macrophage; dendritic cell. |
| **Search execution** | Core query | The ocular inflammation block was combined with the myeloid-cell block using AND. Synonyms within each block were combined using OR. Controlled vocabulary was used where available, together with title, abstract, and keyword terms. |
| **Database-specific search** | PubMed/MEDLINE | ("Uveitis"[Mesh] OR uveitis[tiab] OR "non-infectious uveitis"[tiab] OR "noninfectious uveitis"[tiab] OR "autoimmune uveitis"[tiab] OR "experimental autoimmune uveitis"[tiab] OR "experimental autoimmune uveoretinitis"[tiab] OR uveoretinitis[tiab] OR "ocular inflammation"[tiab]) AND ("Macrophages"[Mesh] OR "Microglia"[Mesh] OR "Monocytes"[Mesh] OR "Myeloid Cells"[Mesh] OR "Dendritic Cells"[Mesh] OR macrophage*[tiab] OR microglia*[tiab] OR monocyte*[tiab] OR myeloid[tiab] OR hyalocyte*[tiab] OR (perivascular[tiab] AND macrophage*[tiab]) OR (choroid*[tiab] AND macrophage*[tiab]) OR (uveal[tiab] AND macrophage*[tiab]) OR (iris[tiab] AND macrophage*[tiab]) OR ("ciliary body"[tiab] AND macrophage*[tiab])) AND english[Language] AND ("1000/01/01"[Date - Publication] : "2026/08/15"[Date - Publication]) |
| **Database-specific search** | Embase | ('uveitis'/exp OR uveitis:ti,ab,kw OR 'non-infectious uveitis':ti,ab,kw OR 'noninfectious uveitis':ti,ab,kw OR 'autoimmune uveitis':ti,ab,kw OR 'experimental autoimmune uveitis':ti,ab,kw OR 'experimental autoimmune uveoretinitis':ti,ab,kw OR uveoretinitis:ti,ab,kw OR 'ocular inflammation':ti,ab,kw) AND ('macrophage'/exp OR 'microglia'/exp OR 'monocyte'/exp OR 'myeloid cell'/exp OR 'dendritic cell'/exp OR macrophage*:ti,ab,kw OR microglia*:ti,ab,kw OR monocyte*:ti,ab,kw OR myeloid:ti,ab,kw OR hyalocyte*:ti,ab,kw OR (perivascular:ti,ab,kw AND macrophage*:ti,ab,kw) OR (choroid*:ti,ab,kw AND macrophage*:ti,ab,kw) OR (uveal:ti,ab,kw AND macrophage*:ti,ab,kw) OR (iris:ti,ab,kw AND macrophage*:ti,ab,kw) OR ('ciliary body':ti,ab,kw AND macrophage*:ti,ab,kw)) AND [english]/lim. Publication-date coverage was limited to database inception through 15 August 2026 in the database interface. |
| **Database-specific search** | Scopus | TITLE-ABS-KEY (uveitis OR "non-infectious uveitis" OR "noninfectious uveitis" OR "autoimmune uveitis" OR "experimental autoimmune uveitis" OR "experimental autoimmune uveoretinitis" OR uveoretinitis OR "ocular inflammation") AND TITLE-ABS-KEY (macrophag* OR microglia* OR monocyte* OR myeloid OR hyalocyte* OR (perivascular W/2 macrophag*) OR (choroid* W/2 macrophag*) OR (uveal W/2 macrophag*) OR (iris W/2 macrophag*) OR ("ciliary body" W/3 macrophag*) OR "dendritic cell*") AND LIMIT-TO (LANGUAGE, "English") AND PUBYEAR < 2027. Results were restricted to records available through 15 August 2026. |
| **Database-specific search** | Web of Science Core Collection | TS=(uveitis OR "non-infectious uveitis" OR "noninfectious uveitis" OR "autoimmune uveitis" OR "experimental autoimmune uveitis" OR "experimental autoimmune uveoretinitis" OR uveoretinitis OR "ocular inflammation") AND TS=(macrophag* OR microglia* OR monocyte* OR myeloid OR hyalocyte* OR "perivascular macrophage*" OR "choroidal macrophage*" OR "uveal macrophage*" OR "iris macrophage*" OR "ciliary body macrophage*" OR "dendritic cell*") AND LA=(English) AND PY=(1900-2026). Results were restricted to records available through 15 August 2026. |
| **Targeted searches** | Cell origin, niche, and profiling | The core query was combined with one or more of the following terms: ontogen*; lineage*; fate mapping; resident; recruited; single cell; single-cell; scRNA-seq; spatial transcriptom*; aqueous humor; vitreous; retina*; choroid*; iris; ciliary body; retinal vessel*; blood-retinal barrier; blood-aqueous barrier. |
| **Targeted searches** | Disease stage and function | The core query was combined with one or more of the following terms: homeostasis; priming; initiation; infiltration; recruitment; extravasation; antigen presentation; amplification; tissue injury; resolution; repair; chronic*; relapse; barrier dysfunction; vascular permeability; phagocytosis. |
| **Targeted searches** | Inflammatory and metabolic pathways | The core query was combined with one or more of the following terms: cytokine*; chemokine*; inflammasome*; NLRP3; AIM2; NLRP12; pyroptosis; P2X7; purinergic; reactive oxygen species; glycolysis; immunometabolism; lactylation; HIF-1alpha; NAMPT; AP-1; trained immunity; innate memory. |
| **Targeted searches** | Therapeutic pathways | The core query was combined with one or more of the following terms: therap*; treat*; clinical trial; inhibitor*; blockade; TNF; IL-6; IL-1; JAK; STAT; CCR2; CXCR3; CSF1R; P2X7; inflammasome; metabolic reprogramming; tissue repair. |
| **Additional identification** | Known-item checks | Exact DOI-, PMID-, and title-directed searches were used for potentially relevant records identified during peer review, bibliographic checking, or examination of closely related literature. These records were evaluated using the same eligibility and evidence-provenance criteria as database-identified records. |
| **Eligibility** | Human studies | Included studies of patients with NIU that examined ocular tissue or fluid, peripheral immune cells linked to ocular disease, cell-resolved or single-cell profiles, functional assays, longitudinal observations, treatment responses, or interventional outcomes relevant to macrophage-lineage biology. |
| **Eligibility** | Experimental studies | Included active-immunization, adoptive-transfer, spontaneous, or other relevant uveitis models that examined macrophages, microglia, monocytes, hyalocytes, uveal macrophages, perivascular macrophages, or interacting myeloid populations. Genetic, depletion, pharmacological, temporal, spatial, and descriptive studies were eligible. |
| **Eligibility** | Therapeutic evidence | Included clinical or experimental studies of targets relevant to macrophage recruitment, activation, antigen presentation, cytokine signaling, inflammasome activity, immunometabolism, barrier regulation, resolution, or repair. Clinical benefit was not assumed to be macrophage-specific when the target was expressed across multiple cell types. |
| **Eligibility** | Background and cross-disease literature | General reviews and studies from other retinal, neuroinflammatory, or immune-mediated diseases were retained only when they provided necessary biological context. They were labeled as background or cross-disease evidence and were not treated as direct NIU mechanisms. |
| **Exclusion** | Records not used as direct evidence | Studies were not used as direct NIU evidence when they focused primarily on infectious uveitis, lacked an ocular inflammatory context, contained no relevant myeloid-cell information, or did not provide sufficient information to evaluate the cited claim. Non-English records and duplicate reports of the same study were not retained as separate evidence units. |
| **Study prioritization** | Priority order | Priority was given to original studies using human ocular tissue or fluid, prospectively characterized patient cohorts, randomized or interventional clinical studies, lineage- or cell-resolved animal experiments, depletion or genetic perturbation, and single-cell or spatial datasets. Reviews were used mainly for background synthesis and literature orientation. |
| **Data charting** | Items recorded | For each study, the authors considered the disease phenotype, species or population, ocular or systemic compartment, myeloid population, disease stage, experimental model, intervention or perturbation, principal outcome, evidence provenance, causal limitations, and therapeutic maturity where applicable. |
| **Evidence provenance** | H-I | Human intervention: randomized, controlled, or other interventional evidence establishing a clinical effect. Such evidence does not by itself identify the responsible myeloid population. |
| **Evidence provenance** | H-D | Human-derived evidence: patient tissue, ocular fluid, blood, single-cell, spatial, observational, or functional data demonstrating a disease-associated state or relationship without necessarily establishing causality. |
| **Evidence provenance** | M-C | Causal uveitis-model evidence: genetic deletion, cell-selective manipulation, depletion, adoptive transfer, pharmacological perturbation with appropriate controls, or another experiment designed to test mechanism in a uveitis model. |
| **Evidence provenance** | M-A | Associative uveitis-model evidence: temporal, spatial, transcriptomic, histological, phenotypic, or correlative observations in a uveitis model without a cell- or pathway-specific causal test. |
| **Evidence provenance** | X | Cross-disease contextual evidence: findings from other ocular, retinal, neurological, or inflammatory conditions used to support plausibility or define a research hypothesis, but not treated as direct evidence of an NIU mechanism. |
| **Therapeutic maturity** | Established clinical use | Regulatory approval or established clinical efficacy in selected forms of NIU, while retaining any limitation in cell specificity. |
| **Therapeutic maturity** | Clinical signal or context-dependent evidence | Human interventional or observational findings indicating potential benefit, with incomplete phase 3, replication, phenotype-specific, or cell-specific support. |
| **Therapeutic maturity** | Preclinical prioritization | Mechanistically supported in uveitis models or exploratory human datasets but without adequate controlled clinical validation in NIU. |
| **Therapeutic maturity** | Hypothesis or repair strategy | A biologically plausible state-modulation or reparative approach that requires target validation, delivery studies, and safety assessment before clinical prioritization. |
| **Claim assessment** | Claim-to-reference mapping | Mechanistically distinct claims were separated where necessary, and citations were placed adjacent to the statement they support. Each claim was interpreted according to the sampled compartment, experimental model, intervention, and level of human support. |
| **Synthesis** | Narrative integration | Evidence sources were treated as complementary dimensions rather than combined into a single ordinal score. Table 1 pairs each major finding with the inference supported by the evidence and the principal unresolved validation gap. |
| **Synthesis** | Quantitative pooling | Meta-analysis was not performed because the included diagnoses, anatomical compartments, experimental models, interventions, outcome measures, and sampling designs were too heterogeneous for meaningful quantitative pooling. |
| **Author review** | Final decisions | The authors determined final eligibility, evidence-provenance labels, interpretation, and manuscript inclusion. AI-assisted tools were not used to determine study eligibility or independently interpret primary data. |
